# Acceptance of Teledermatological Practices: A Cross-Sectional Study of Practicing Saudi Dermatologists

**DOI:** 10.7759/cureus.13710

**Published:** 2021-03-05

**Authors:** Abdullah Alakeel

**Affiliations:** 1 Dermatology Department, College of Medicine, King Saud University, Riyadh, SAU

**Keywords:** telemedicine, dermatology, saudi arabia

## Abstract

Background: The increase in demand for Telemedicine globally has continued to rise in recent months, showing remarkable success in a variety of medical fields. As dermatology shows one of the most prolific rates of success, having a clear national picture of practitioner opinion on the subject is important. In this study we aimed to quantify the overall level of acceptance and satisfaction of Saudi dermatologists towards the practice of teledermatology.

Design and setting: Data was collected by means of a survey which included demographic data and level of exposure and overall satisfaction towards Telemedicine in their field.

Results: A total of 102 questionnaires were returned of which 57.8% had engaged in at least one teleconsultation. Of these, 71.2% showed support for the technology, with negative responses being associated with poor infrastructure or implementation in the workplace.

Conclusion: The acceptance of dermatologists to teledermatology consultations in Saudi Arabia shows a positive tendency, with the majority of those who engaged in the practice saying they would do so again and find it satisfactory. Creating more reliable and robust tools and greater training in their use would be beneficial for its further incorporation.

## Introduction

The practice of telemedicine is drastically changing healthcare services delivery due to its flexibility and easy integration into a multitude of areas and specialties [1]. As an emerging practice, telemedicine is loosely described as a medical consultation provided via a remote channel, usually via video-conference software and associated equipment [2,3] in which the most salient attributes are cost-effectiveness for both parties and overall emphasis on comfort and privacy afforded by avoiding public facilities [4]. 

Dermatology and its various conditions are commonly primarily visually assessed by a dermatologist. As telemedicine is a purely visual medium of communication, it has potential of achieving the highest rates of adoption and acceptance worldwide [5]. In a single teleconsultation, an assessing physician may, by means of instructions and detailed questioning of symptoms and medical history, effectively and accurately diagnose a significant number of conditions and pathologies [6]. This can be further augmented by the ability to prescribe treatment and issue follow-ups without any need for intermediaries or delays [7].

The global level of satisfaction towards teledermatology is not homogenous, however. Although its adoption in many parts of the globe is relatively high, there is still some resistance by physicians in certain areas, most notably developing countries [8,9]. Studies into this matter largely attribute this to a technological barrier, as these countries rarely possess the resources and technical knowledge to correctly implement the necessary infrastructures, and qualified staff to raise awareness and teach local practitioners on its uses [10].

Patient views on the practice is of equal importance and, to this end, awareness campaigns and active promoting of the service and its benefits to the individual have been conducted in many countries [11]. The overall result of these efforts has been a visible increase in levels of adoption and satisfaction on part of patients, which has been shown to closely correlate with their level of understanding of the service [12,13].

This study was conducted with the aim of determining the relative level of acceptance and satisfaction of telemedicine in the eyes of practicing dermatologists which reside and conduct their work in Saudi Arabia.

## Materials and methods

Study design

This was a cross-sectional qualitative study carried out in Saudi Arabia. Only licensed and actively practicing dermatologists were taken into consideration when gathering data.

Data collection

A self-administered survey was distributed after validation to as many dermatologists as possible, containing questions on basic demographic information, practical experience in the field, and questions on telemedicine participation history and degree of approval. These were supplemented by questions on the reasons behind their level of approval and what they considered to be the limiting factors for further increase. The possible answers to these questions varied from Strongly Disagree to Strongly Agree at the ends of the spectrum, and Slightly Agree/Disagree and Neutral as the other three possible stances (see the questionnaire in the appendix).

Statistical analyses

Demographic categorical data were expressed as percentages. Numerical scores were expressed in terms of their means ± sd were used to describe numerical variables. To assess whether a positive response to the questions on satisfaction relating to the practice, systems and patient acceptance of telemedicine had any correlation with demographic variables, each response above neutral was attributed a score of 1, and the sum for each separate question was used as a comparison basis using a one-way analysis of variance (ANOVA).

Statistical significance was set at p-value<0.05. The software package used for testing was Statistical Package for Social Sciences (SPSS) version 26 (IBM Corp., Armonk, NY, USA).

Validation of the questionnaire

Validation of the questions included in this questionnaire was carried out through Cronbach’s alpha test. Validation for each section was performed separately followed by validation of the questionnaire as a whole. It has been shown that Cronbach’s alpha for attitude section was 0.707.

Ethical considerations

Institutional ethics board approval was acquired before conducting any study procedure. Dermatologists were informed that their participation was entirely voluntary, and that all information was made anonymous and maintained strictly as confidential.

## Results

A total of 102 questionnaires were returned and used as the base sample size (n=102). Demographic data can be viewed below. Of all the demographic information, only gender could be seen to have a significant bias towards male practitioners, comprising 70% of the total sample (Table 1).

**Table 1:** Summary of demographic qualifying data of participants. n=102

		Count	Percent
Gender	Male	70	68.6
	Female	32	31.4
Years of experience	1 to 3 years	31	30.4
	4 to 6 years	7	6.9
	6 to 10 years	25	24.5
	More than ten years	39	38.2
Have you practiced a full teledermatology consultation?	Yes	59	57.8
	No	43	42.2

Of the 102 participants, a total of 59 (57.8%) had engaged in at least one instance of telemedicine comprise the sample size for any questions relating to service views and overall satisfaction.

Reasons for not practicing tele-dermatological consultations

**Figure 1:**
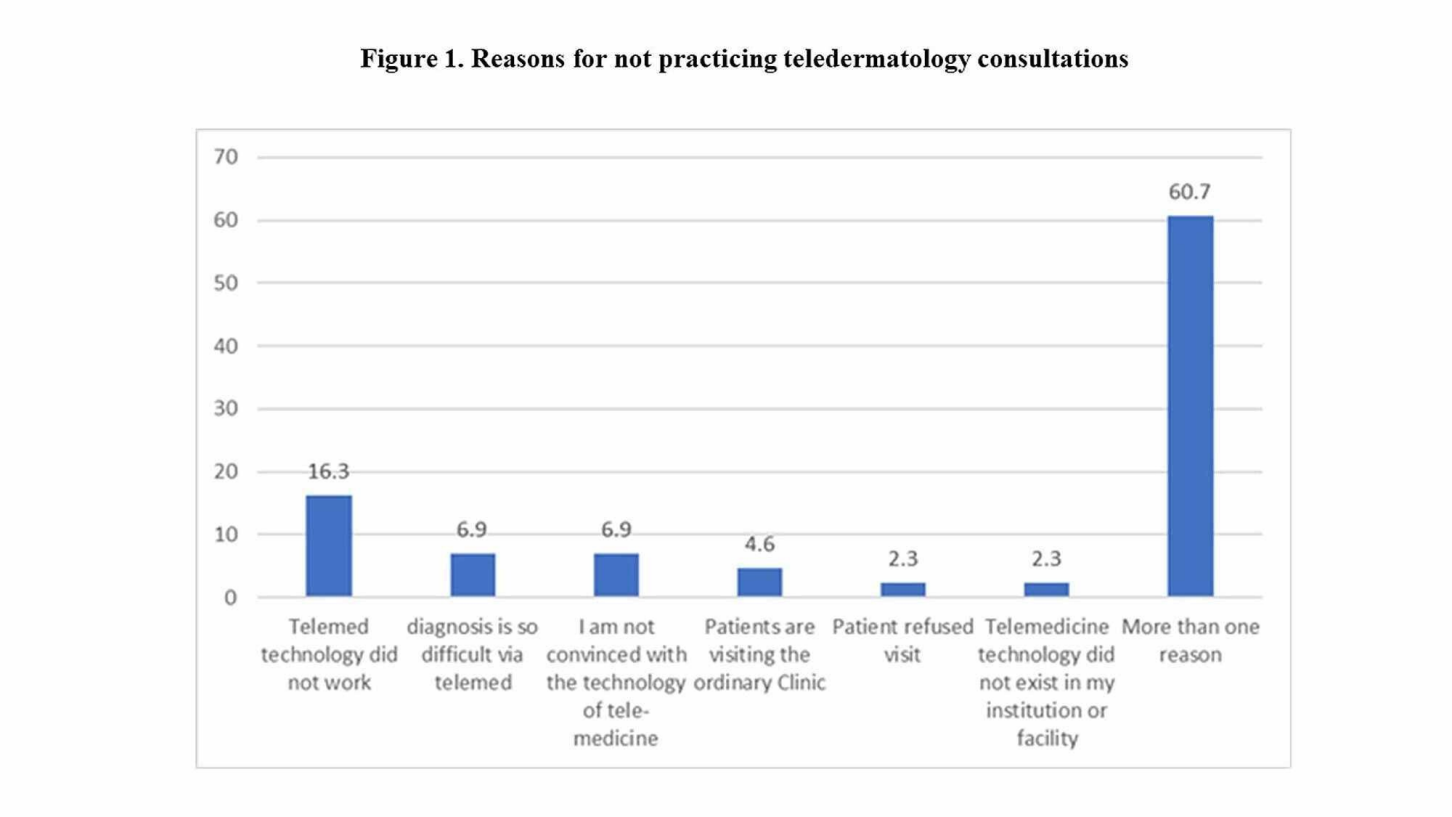
Reasons given for not having engaged in telemedicine. n=43

Of the 43 participants that responded as having never engaged in telemedicine, the most common reason (16.3%) given was a distrust or experienced failure in the technology necessary for its practice. The least common reasons were equally patient refusal to participate and lack of proper facilities, both at 2.3%. The majority (60.7%) of this sample group gave multiple reasons for not engaging in telemedicine (Figure 1).

Assessment of demographic data on engagement in telemedicine

Analysis on whether gender or years of practice had any effect on whether the participant had engaged in a telemedicine consultation found no correlation for either group (p-value=0.282; p-value=0.307 respectively) (Table 2).

**Table 2:** Comparison between practitioners and non-practitioners. n=102

		Have you practiced a full teledermatology consultation?		p-value
		No	Yes	
Gender	Male	74.4%	64.4%	0.282
	Female	25.6%	35.6%	
Years of experience	1 to 3 years	23.3%	35.6%	0.307
	4 to 6 years	9.3%	5.1%	
	6 to 10 years	20.9%	27.1%	
	More than 10 years	46.5%	32.2%	

Opinions of dermatologists who practiced telemedicine

Of the 59 participants in this category, most responses were of the positive category (either Strongly agree or Agree) or the neutral category. The negative categories never comprised more than 11.9% of the total, most remaining around 5% or less (Table 3).

**Table 3:** Opinions of dermatologists towards telemedicine consultations

		Count	Percent
I would use the technology for patient care and management frequently and routinely	Strongly agree	12	20.3
	agree	22	37.3
	neutral	18	30.5
	disagree	6	10.2
	Strongly disagree	1	1.7
I would use telemedicine when it becomes available in my departments or hospitals	Strongly agree	17	28.8
	agree	23	39.0
	neutral	17	28.8
	disagree	1	1.7
	Strongly disagree	1	1.7
I am qualified to use telemedicine technology for clinical purposes	Strongly agree	19	32.2
	agree	27	45.8
	neutral	11	18.6
	disagree	2	3.4
	Strongly disagree	0	0
telemedicine consultation may have assessed the patient’s outcome	Strongly agree	12	20.3
	agree	27	45.8
	neutral	18	30.5
	disagree	1	1.7
	Strongly disagree	1	1.7
telemedicine consultation success depends on clinical decision making	Strongly agree	10	16.9
	agree	27	45.8
	neutral	19	32.2
	disagree	3	5.1
	Strongly disagree	0	0

Overall satisfaction of dermatologists with telemedical practices

Of the 59 participants who had engaged in telemedicine, over half were expressed satisfaction with the outcome (65.9%), the system framework and reliability (64.4%), and with reported patient satisfaction (78%). The number of negative or neutral responses was always of negligible amount (Table 4).

**Table 4:** The satisfaction of dermatologists with telemedicine

		Count	Percent
Based on your perception of telemedicine consultation, how satisfied are you with telemedicine outcome?	Completely satisfied	5	8.5
	Generally satisfied	34	57.6
	neutral	15	25.4
	Generally unsatisfied	5	8.5
	Completely unsatisfied	0	0
Based on your perception, what is your overall satisfaction with the telemedicine system for this telemedicine consultation?	Completely satisfied	5	8.5
	Generally satisfied	33	55.9
	neutral	10	16.9
	Generally unsatisfied	11	18.6
	Completely unsatisfied	0	0
What is your perception of overall patient satisfaction with telemedicine consultation?	Completely satisfied	8	13.6
	Generally satisfied	38	64.4
	neutral	13	22.0
	Generally unsatisfied	0	0
	Completely unsatisfied	0	0

COVID-19 as a factor on response

**Figure 2:**
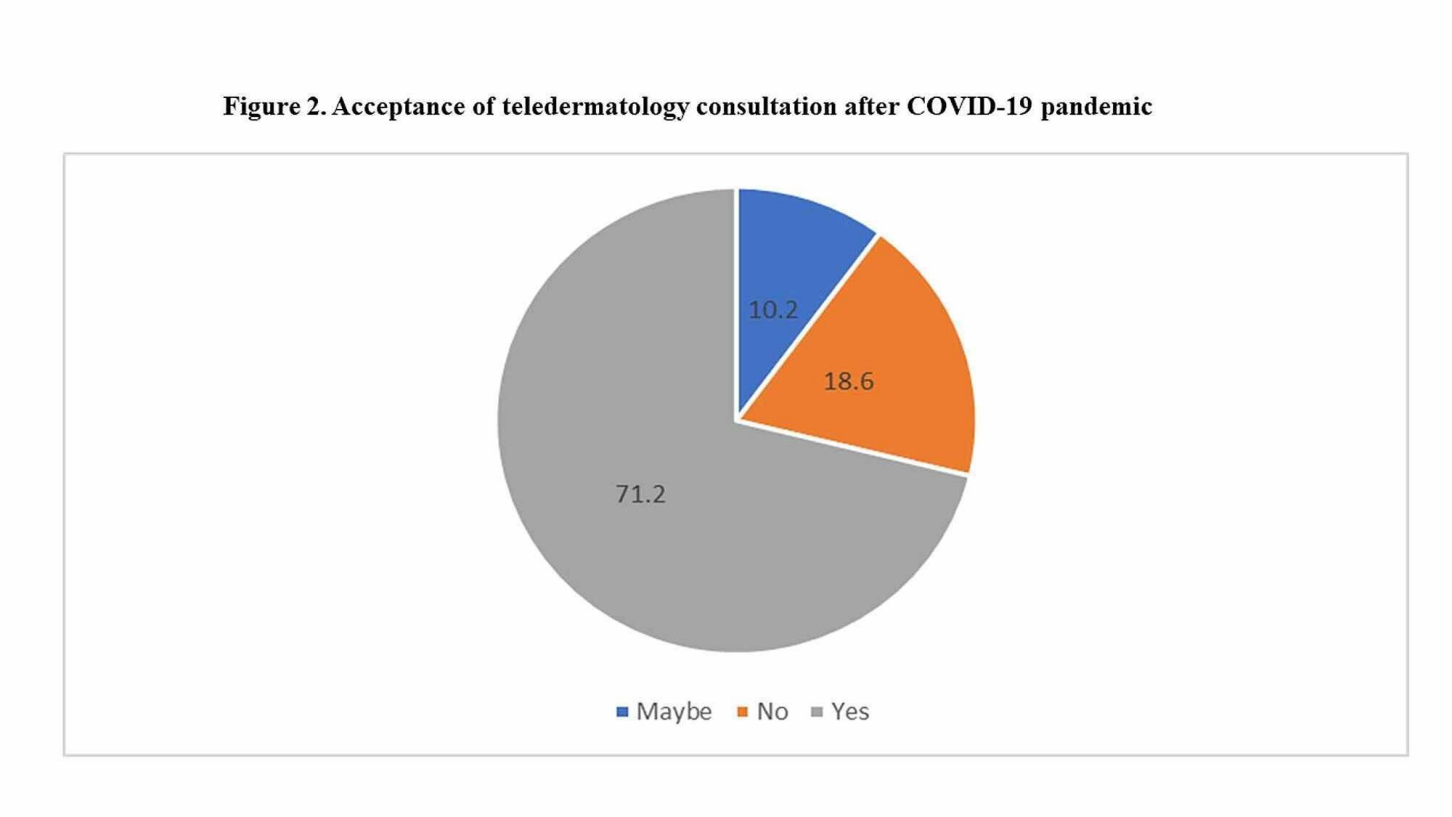
COVID-19 as a factor on participant responses

When asked if the current coronavirus disease 2019 (COVID-19) pandemic had affected their acceptance and overall opinion of telemedicine, 71.2% agreed that their views were affected in some way by the current pandemic, with the remaining 18.6% stating it had had no bearing on their responses (Figure 2).

Assessing positive response with demographic variables

The average score for positive responses was 1.3±1.9, ranging from 0 to 7. No significant difference between acceptance and demographics was found for any of the variables (Table 5).

**Table 5:** Comparison of acceptance score among different demographic variables

		Count	Mean	SD	Minimum	Maximum	P-value
Age category	Less than 30	17	1.7	2.2	0.0	7.0	0.418
	30 to 40	32	1.3	1.8	0.0	6.0	
	40 to 50	8	0.6	1.2	0.0	3.0	
	50 to 60	2	0.0	0.0	0.0	0.0	
Gender	Male	38	1.0	1.6	0.0	6.0	0.088
	Female	21	1.9	2.2	0.0	7.0	
Years of experience	1 to 3 years	21	1.7	2.3	0.0	7.0	0.635
	4 to 6 years	4	1.3	2.5	0.0	5.0	
	6 to 10 years	15	0.9	1.5	0.0	4.0	
	More than ten years	19	1.2	1.5	0.0	4.0	

Questionnaire validation testing

Results for attitude, satisfaction and overall questionnaire validity are detailed below, with each category and its related questions being summarized separately (Tables 6-8).

**Table 6:** Cronbach’s alpha for attitude questions

	Cronbach's Alpha if Item Deleted
[I would use the technology for patient care and management frequently and routinely]	0.737
[I would use telemedicine when it becomes available in my departments or hospitals]	0.486
[I am qualified to use telemedicine technology for clinical purposes]	0.686
[telemedicine consultation may have assessed the patient’s outcome]	0.583
[telemedicine consultation ‘success depend on clinical decision making]	0.721

**Table 7:** Cronbach’s alpha for satisfaction questions

	Cronbach's Alpha if Item Deleted
[Based on your perception about telemedicine consultation, how satisfied are you with telemedicine outcome?]	0.889
[Based on your perception, what is your overall satisfaction with the telemedicine system for this telemedicine consultation?]	0.765
[What is your perception about overall patient satisfaction with telemedicine consultation?]	0.629

**Table 8:** Cronbach’s alpha for the whole questionnaire

	Cronbach's Alpha if Item Deleted
[I would use the technology for patient care and management frequently and routinely]	0.712
[I would use telemedicine when it becomes available in my departments or hospitals]	0.631
[I am qualified to use telemedicine technology for clinical purposes]	0.684
[telemedicine consultation may have assessed the patient’s outcome]	0.675
[telemedicine consultation ‘success depend on clinical decision making]	0.766
[Based on your perception about telemedicine consultation, how satisfied are you with telemedicine outcome?]	0.753
[Based on your perception, what is your overall satisfaction with the telemedicine system for this telemedicine consultation?]	0.667
[What is your perception about overall patient satisfaction with telemedicine consultation?]	0.669

## Discussion

Digital technologies are increasingly prevalent in medicine as they have been shown to have a mostly positive impact on patient care and overall outcome [14]. This gradual integration was drastically accelerated by the COVID-19 pandemic of 2020 which forced medical practices to adapt to a world where face-to-face consultation was not legally permissive unless it in emergency situations [15]. Telemedicine in particular has seen a rapid increase in implementation on a global scale, and dermatology, relying mostly on visual assessment for diagnosis, ranks highly among those adopting the practice [16]. Whether or not the physicians themselves approve of this integration is still not clear, however [17]. It was in view of assessing these views that this study was conducted.

Similar to other studies [18-20], we found that telemedicine is indeed seeing a high level of adoption and overall acceptance in the field of dermatology, with over half the participants stating they had engaged in a session at least once. Of those who hadn’t, the reasons given were mostly related to technical limitations such as insufficient infrastructures or unreliable tools and software platforms, and not the poor perception of telemedicine as an addition to available medical practices.

When assessing demographic variables on the level of acceptance, no meaningful relation or correlation could be established, indicating that whatever opinion the participant expressed, it was not influenced by their age, gender or how long they had practiced in their field. This is encouraging as it suggests that possible stereotypical pressures on expected behaviors and responses were not a meaningful factor, and that other factors must therefore be responsible.

When reviewing similar studies for possible reasons for acceptance or distrust of telemedicine, one possible factor shown to have a significant impact was level of awareness of physicians concerning the technology, its applications, and its benefits [18,19].

It is of interest that most participants stated that the current pandemic had a decided impact on their views and adoption of telemedicine as a medical practice. It will be of considerable interest to see if this remains the same after the situation is resolved.

The findings of Trettel et al. [18] support the outcomes of the present study, where the acceptance rate of teledermatology was relatively low, with an average score of 1.3±1.9. Additionally, only 57.8% had practiced a full teledermatology consultation previously, while dermatologists who did not practice teledermatology were mainly because of the service's absence or it did not work.

## Conclusions

The practice of telemedicine applied to dermatology in Saudi Arabia is increasing at a rapid pace and, overall, the perception and opinions for its future as a tool in medical practice is a positive one. The lack of infrastructure and awareness are indicated as the areas in which improvements can be made to further this integration. An assessment into whether these findings remain true after the pandemic would be of great value in assessing how its effect on daily life may have impacted long-established practices and beliefs 

Limitations

The present study suffered from a relatively small sample size, a factor that was considered during data interpretation. Furthermore, the study was entirely reliant on participant honesty and objectivity in answering the questions as there was no way to guarantee or monitor these factors. It is therefore advised that appropriate caution and critical assessment be used when interpreting our findings.

## Appendices

A survey of dermatologist’s acceptance of telemedicine:

1.     Age: …………………………………………………………………..

2.     Sex:

o   Male

o   Female

3.     Years of experience:

o   1-3

o   4-6

o   6-10

o   >10

4.     Do you attend a complete telemedicine visit?

o   Yes

o   No

5.     IF no, why?

o   Telemed technology did not work

o   Patient refused visit

o   Patient no show

o   Clinician unavailable

o   Clinic or session cancelled

o   Clinic or session occurred

o   Questions related to acceptance

**Table 9:** final part of the questionnaire

	Strongly agree	agree	neutral	disagree	Strongly disagree
I would use the technology for patient care and management frequently and routinely					
I would use telemedicine when it became available in my departments or hospitals					
I am qualified to use telemedicine technology for clinical purposes					
telemedicine visit may have improved the patient’s prognosis					
telemedicine visit ‘ success depend on clinical decision making					
	Completely satisfied	Generally satisfied	Neutral	Generally unsatisfied	Completely unsatisfied
Based on your perception about telemedicine visit, how satisfied are you with telemedicine outcome?					
Based on your perception, what is your overall satisfaction with the telemedicine system for this telemedicine visit?					
What is your perception about overall patient satisfaction with telemedicine visit?					
